# Targeting PCSK9 as a key player in lipid metabolism: exploiting the therapeutic and biosensing potential of aptamers

**DOI:** 10.1186/s12944-024-02151-8

**Published:** 2024-05-25

**Authors:** Maryam Mahjoubin-Tehran, Samaneh Rezaei, Raul D. Santos, Tannaz Jamialahmadi, Wael Almahmeed, Amirhossein Sahebkar

**Affiliations:** 1 Biotechnology Research Center, Pharmaceutical Technology Institute, Mashhad University of Medical Sciences, Mashhad, Iran; 2 Department of Medical Biotechnology and Nanotechnology, Faculty of Medicine, Mashhad University of Medical Sciences, Mashhad, Iran; 3 Lipid Clinic Heart Institute (Incor), University of São Paulo, Medical School Hospital, São Paulo, Brazil; 4 Pharmaceutical Research Center, Pharmaceutical Technology Institute, Mashhad University of Medical Sciences, Mashhad, Iran; 5 Medical Toxicology Research Center, Mashhad University of Medical Sciences, Mashhad, Iran; 6 Heart and Vascular Institute, Cleveland Clinic Abu Dhabi, Abu Dhabi, United Arab Emirates; 7 Applied Biomedical Research Center, Mashhad University of Medical Sciences, Mashhad, Iran

**Keywords:** Aptamer, PCSK9, LDLR, LDL, Hypercholesterolemia

## Abstract

The degradation of low-density lipoprotein receptor (LDLR) is induced by proprotein convertase subtilisin/kexin type 9 (PCSK9), resulting in elevated plasma concentrations of LDL cholesterol. Therefore, inhibiting the interactions between PCSK9 and LDLR is a desirable therapeutic goal for managing hypercholesterolemia. Aptamers, which are RNA or single-stranded DNA sequences, can recognize their targets based on their secondary structure. Aptamers exhibit high selectivity and affinity for binding to target molecules. The systematic evolution of ligands by exponential enrichment (SELEX), a combination of biological approaches, is used to screen most aptamers in vitro. Due to their unique advantages, aptamers have garnered significant interest since their discovery and have found extensive applications in various fields. Aptamers have been increasingly utilized in the development of biosensors for sensitive detection of pathogens, analytes, toxins, drug residues, and malignant cells. Furthermore, similar to monoclonal antibodies, aptamers can serve as therapeutic tools. Unlike certain protein therapeutics, aptamers do not elicit antibody responses, and their modified sugars at the 2’-positions generally prevent toll-like receptor-mediated innate immune responses. The focus of this review is on aptamer-based targeting of PCSK9 and the application of aptamers both as biosensors and therapeutic agents.

## Introduction

Cardiovascular disease (CVD) is one of the most prevalent illnesses in industrialized nations and accounts for a considerable rate of annual deaths [1, 2]. Atherosclerosis is a primary cause of CVD, and elevated plasma concentrations of low-density lipoprotein cholesterol (LDL-C) play a pivotal role in CVD development and progression. People with familial hypercholesterolemia, a genetic disease characterized by a reduction in plasma clearance of LDL, develop premature atherosclerosis [3, 4] and have a greater risk of mortality than does the general population. Additionally, there is a causal correlation between plasma LDL-C concentrations and the risk of coronary heart disease (CHD): a 0.8 mmol/L (30 mg/dL) increase in plasma LDL-C results in a 30% increase in CHD risk [5, 6]. Therefore, reducing plasma LDL-C levels can help prevent atherosclerosis and lower CVD risk [7].

Statin therapy is commonly used to treat hypercholesterolemia by inhibiting hepatic cholesterol production, increasing the expression of LDL receptors (LDLRs) and enhancing LDL clearance from plasma, thereby reducing CVD risk. In addition, statins have many pleiotropic effects [8–18], particularly anti-inflammatory and antioxidant effects [19–24]. However, many individuals using statins fail to achieve the proposed LDL-C goals [25], and a considerable number of patients may not tolerate these drugs [26]. This has led to the introduction of several classes of newer LDL-lowering agents [27–30]. The potential of statins to lower plasma LDL-C levels is limited by the fact that statin therapy paradoxically increases both circulating plasma proprotein convertase subtilisin/kexin type 9 (PCSK9) and hepatic LDLR expression [31]. To address the issues associated with statin-based hypercholesterolemia therapy, monoclonal antibodies (mAbs) targeting PCSK9, such as evolocumab and alirocumab, have been developed. In combination with statins, PCSK9-targeting mAbs effectively reduce LDL-C and consequent morbidity and mortality in patients with CVD [32]. However, mAbs have certain limitations, including high manufacturing costs and oral unavailability, which restrict their widespread use [33].

### PCSK9

**Fig. 1:**
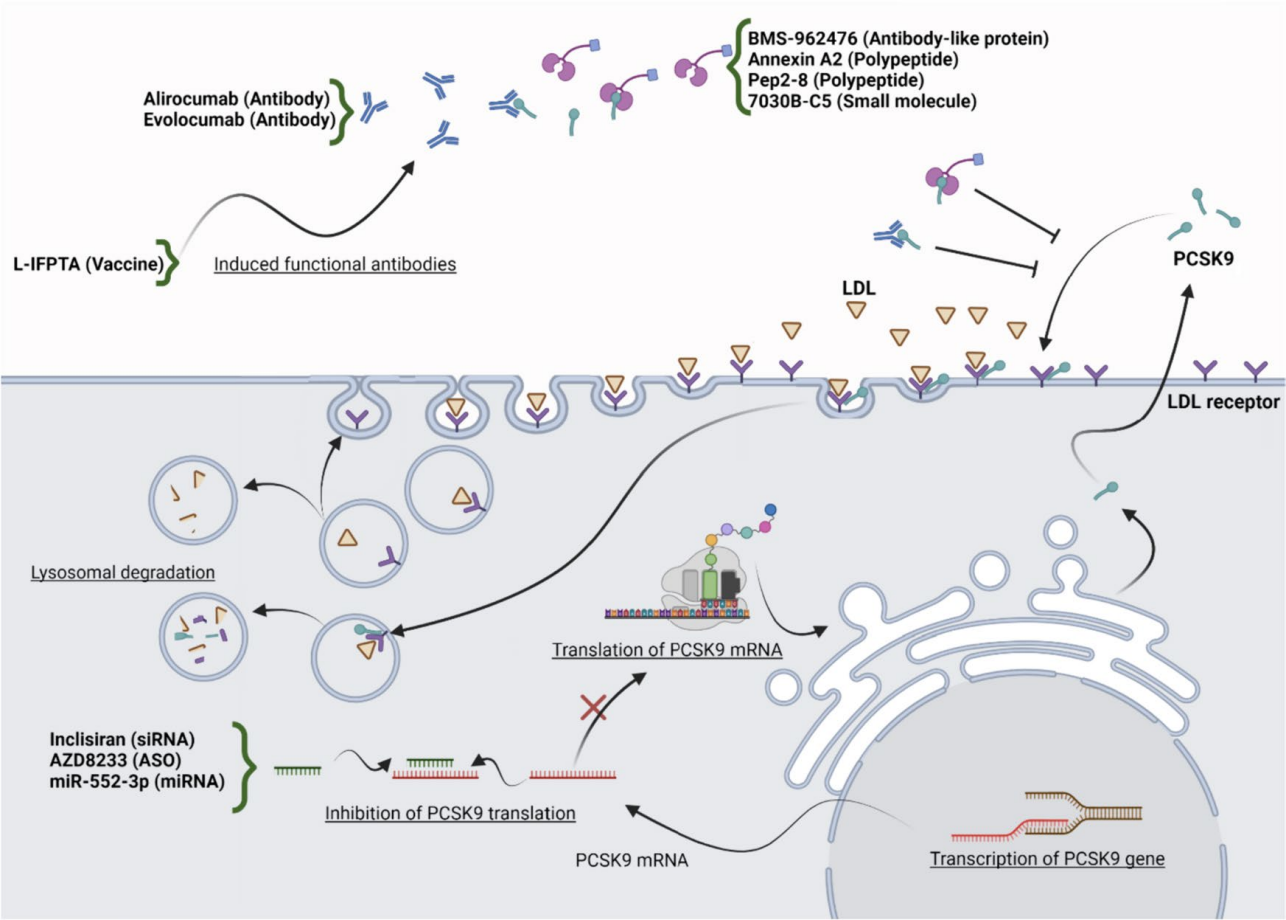
Schematic representation of the role of PCSK9 inhibitor therapeutics in LDL catabolism. After transcription of the PCSK9 gene in the nucleus, PCSK9 is produced via translation in the cytosol. Inclisiran, AZD8233, and MiR-552-3p bind to PCSK9 mRNA and inhibit its translation. The liver secretes PCSK9 after it has matured and undergoes several posttranslational changes. PCSK9 binds to LDLR, and LDLR is degraded in the lysosome as a result of the PCSK9-LDLR interaction, which increases plasma LDL-C levels while lowering hepatic LDLR expression. Alirocumab, evolocumab, BMS-962,476, Annexin A2, Pep2-8, and 7030B-C5 bind to PCSK9 and prevent the PCSK9-LDLR interaction. L-IFPTA as a vaccine induces functional antibody production [41]

PCSK9, primarily released by the small intestine, liver, and kidney, plays a role in LDL-C catabolism by interacting with hepatic LDLRs, which transport LDL-C from the plasma into hepatocytes (Fig. 1). PCSK9 consists of a 30-amino-acid signaling peptide, a catalytic domain, a 14-kDa prodomain, and a C-terminal histidine/cysteine-rich domain [34]. The catabolism of LDL by PCSK9 involves several processes (Fig. 1): (i) PCSK9 is synthesized as a precursor protein via ribosomal protein synthesis in the cytosol; (ii) in the endoplasmic reticulum, PCSK9 undergoes autocatalytic cleavage, producing the prodomain; (iii) the catalytic domain’s active site is tightly bound by the prodomain, rendering it catalytically inactive; (iv) after maturation and various posttranslational modifications, the liver secretes PCSK9; and (v) the interaction between PCSK9 and LDLRs leads to the degradation of LDLRs in the lysosome, resulting in reduced LDLR expression on the hepatocyte surface and consequently elevated plasma LDL-C [35, 36]. Gain-of-function variants in *PCSK9* lead to increased plasma LDL-C concentrations and hypercholesterolemia, while loss-of-function variants are associated with reduced plasma LDL-C and decreased CHD risk. Therefore, PCSK9 represents a therapeutic target for managing hypercholesterolemia [37]. In addition, recent evidence has revealed numerous extrahepatic functions for PCSK9 [38–40].

A number of gain-of-function variants in *PCSK9*, which play a crucial role in maintaining cholesterol homeostasis, result in hypercholesterolemia [42]. Individuals with the severe form of FH have been found to overexpress the *PCSK9* D374Y mutant, which exhibits a greater affinity for LDLRs than does wild-type PCSK9 [43]. FH affects approximately 1 in 300 people worldwide, making it the most common inherited metabolic disorder. However, it is often underdiagnosed and undertreated in the general population, which exacerbates its clinical severity [44].

The limitations of statins in effectively reducing LDL-C levels in certain patients, such as those with FH, have prompted research efforts to discover new therapeutics with different mechanisms of action for managing cholesterol. One therapeutic approach for cholesterol regulation that has been clinically validated is the development of PCSK9 inhibitors (Fig. 1). Monoclonal antibody (mAb) inhibitors, including alirocumab and evolocumab, have received FDA approval for the treatment of high-risk individuals with hypercholesterolemia [37], including FH [45, 46]. Peptides/proteins, such as small molecules such as BMS-962,476, act as inhibitors by preventing the binding of PCSK9 to LDLR. Nucleic acids, such as siRNAs such as inclisiran [47], miRNAs, and antisense oligonucleotides (ASOs), can bind to PCSK9 mRNA and inhibit its translation. Vaccines, such as liposomal immunogenic-fused PCSK9-Tetanus plus alum adjuvant (L-IFPTA), stimulate the production of functional endogenous antibodies [41]. Several natural products with PCSK9-inhibiting activity have also been identified [48].

### Aptamers

Aptamers have recently emerged as analytical tools with the potential to replace antibodies in conjunction with immunoassays, flow cytometry, imaging, and mass spectrometry (MS). They have even been suggested for use in cancer treatment. These molecules are typically selected from a pool of random sequences using a general technique termed systematic evolution of ligands by exponential enrichment (SELEX) [49]. Aptamers possess a nucleotide sequence and tertiary structure that enable them to selectively and strongly bind to specific molecules of interest. Several studies have used modified methods for screening aptamers to improve their efficacy [50]. These molecules can have various conformations and encompass a wide range of targets, including cells, tissues, proteins, nucleic acids, and small molecules [51–54].

Aptamers differ significantly from antibodies, as they exhibit higher selectivity and often possess greater affinities. Aptamers, such as mAbs, can be used for therapeutic purposes. However, unlike traditional methods for producing mABs, the in vitro selection of oligonucleotides does not require the use of organisms. This approach offers numerous advantages in manipulating the process of directed evolution. Furthermore, aptamers do not elicit an antibody response due to their nonimmunogenic nature, and their 2′-modified sugar content helps mitigate toll-like receptor-mediated innate immune responses [51, 54, 55].

Unlike antibodies, aptamers are synthetic, which potentially makes them more cost-effective to produce, more scalable with faster lead times, and less prone to batch-to-batch variability. Their nonimmunogenicity, ease of synthesis, stability, high affinity, and target selectivity make them highly suitable for therapeutic applications. In recent years, research on the medicinal applications of aptamers has rapidly expanded. Since their discovery, the therapeutic potential of aptamers for treating illnesses such as hepatitis, cancer, and HIV has been extensively studied [56–58].

Through combinatorial chemical techniques, numerous aptamers have been discovered in the laboratory, and an increasing number of such materials are now found in nature. While these aptamers often exhibit affinities and activities comparable to those of antibodies, only a few have entered clinical research, in contrast to the vast and growing number of therapeutic antibodies. Macugen (Pegaptanib), an aptamer targeting vascular endothelial growth factor (VEGF), is the only aptamer that has reached the market as a therapeutic agent. However, three others, Fovista (targeting PDGF) and Pegnivacogin (targeting coagulation factor IXa), have reached phase III clinical trials, and Zimura (targeting complement factor C5) has been approved by the FDA [59, 60].

Aptamers also hold potential as high-affinity reagents for MS. Furthermore, it has been recently demonstrated that plasma measurement of PCSK9 is achievable using immunoaffinity enrichment liquid chromatography‒mass spectrometry (LC‒MS) with a SOMAmer containing a single modified nucleotide [61].

From a diagnostic perspective, aptamer-based biosensors, known as aptasensors, utilize aptamers as biorecognition elements. Aptamers exhibit resistance to degradation or denaturation, are easily labeled without loss of function, are small in size, and enable homogeneous assays, facilitating quick and simple testing platforms. Over the past decade, aptamers have been employed in numerous diagnostic platforms for the detection of various analytes, ranging from small molecules to more complex targets [62–64].

### Aptamers for targeting PCSK9

**Fig. 2:**
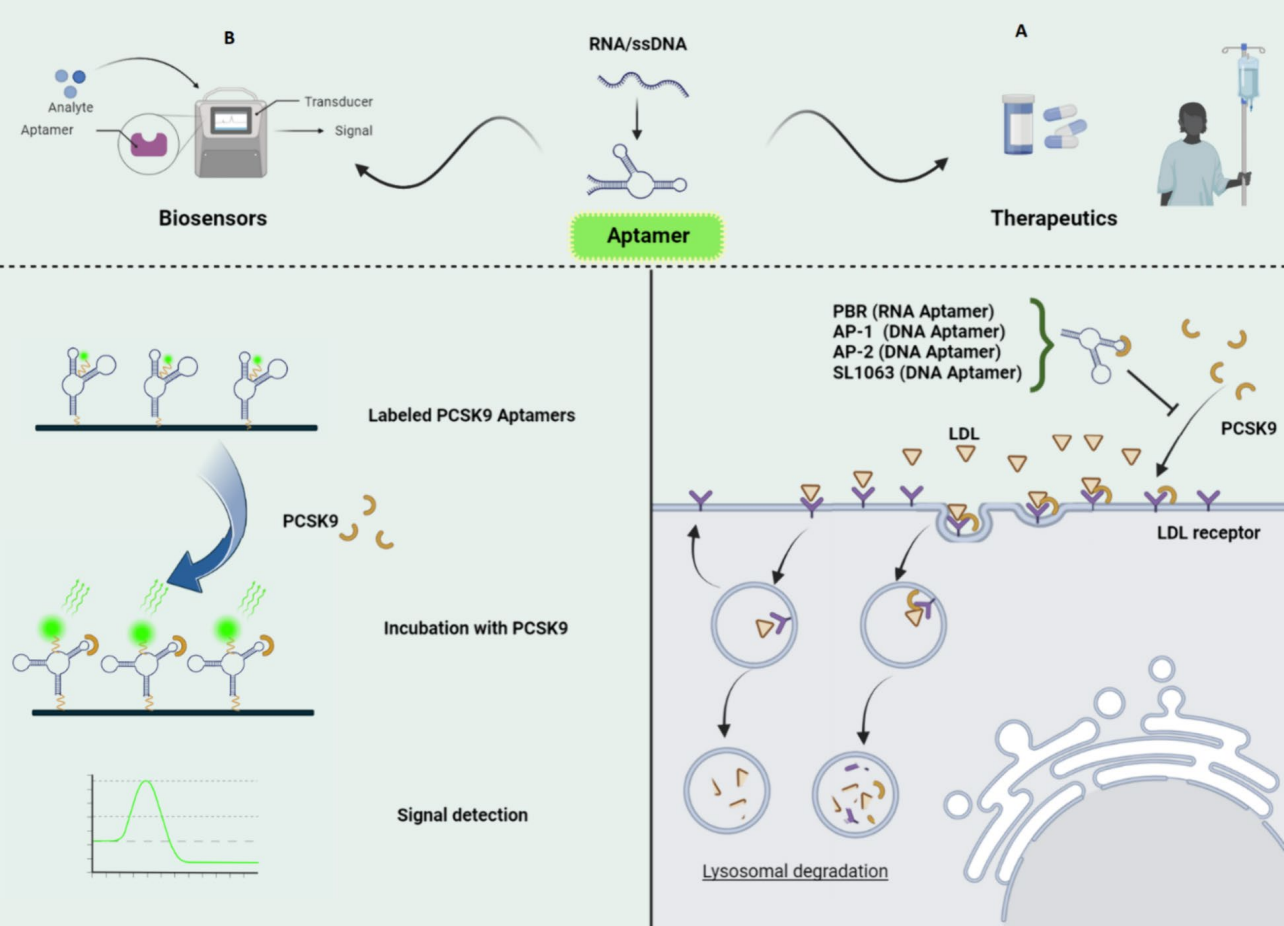
PCSK9 aptamers used for biosensors and targeted therapy. **A** For therapeutics, PCSK9 aptamers such as PBR, AP-1, AP-2, and SL1063 bind to PCSK9 and prevent LDLR-PCSK9 interactions. **B** For biosensor application, labeled PCSK9 aptamers specifically detect the PCSK9 molecules. Binding to PCSK9 causes alterations in the structure of the aptamer, which changes the fluorescence intensity

**Fig. 3:**
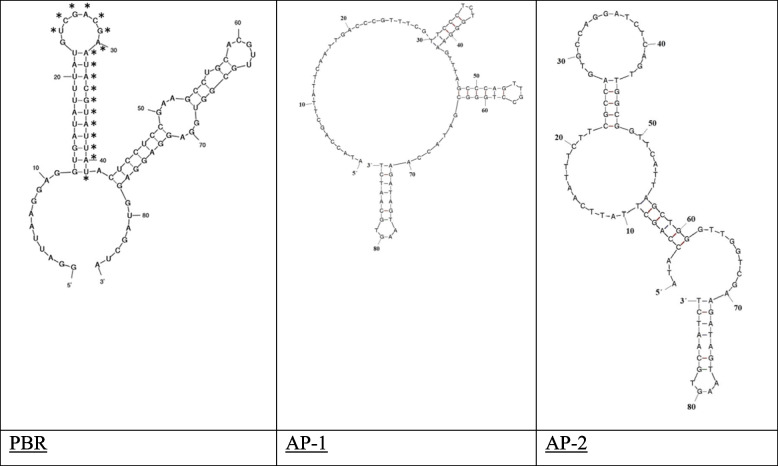
Secondary structure of PCSK9 Aptamers created with the Mfold web server

 Recently, aptamers have been utilized in several studies for targeting PCSK9 in therapeutic or biosensor applications, as described below (Fig. 2). The properties and sequences of these aptamers are listed in Tables 1 and 2, respectively. Additionally, the secondary structures of these aptamers are depicted in Fig. 3.

**Table 1:** Properties of PCSK9 aptamers for therapeutics

Name of Aptamer	Type of Aptamer	ΔG: kcal/mol	IC50^a^	Dissociation constant (KD)^b^	Sequence length	Ref.
PBR	RNA	-	120 nM	70 nM	85	[65]
AP-1	DNA	− 13.06^c^, − 9.17^d^	325 nM	294 nM	88	[66]
AP-2	DNA	− 12.94^c^, − 8.28^d^	327 nM	323 nM	88	[66]
SL1063	DNA	-	2.8 nM (wild-type human PCSK9) 35 pM (D374Y mutant)	wild type, Kd = 14.7 pM; D374Y mutant, Kd = 5.2 pM	30	[61]

^a^IC50: The IC50 value of an aptamer quantifies how much is required to block the interaction of PCSK9 with LDLR by 50%

^b^KD: Dissociation constant. Therefore, if KD is high, the aptamer has poor affinity for PCSK9 and must be present at high concentrations to cover 50% of the protein. The KD was determined at 37 °C. The KD for the interaction of LDLR with PCSK9 was 628-810 nM

^c^At 25 °C

^d^At 37 °C

**Table 2:** Sequence of the PCSK9 aptamers

Name of Aptamer	Sequence	Ref.
PBR	GGAUUAAGGAGGUGAUAUUUAUGUCGACGAAUACGUAUUAUA CUCCUCCGAAGCCUGCACGUUGCGGUGGAGGAGGAGGUAGCUA	[65]
AP-1	ATACCAGCTTATTCAATTGACCCGTTTCGTTCCCTCTGGGAAGT TTAGCCCAGTTGCCTGGGCGATACCAAGATAGTAAGTGCAATCT	[66]
AP-2	ATACCAGCTTATTCAATTTCTTCGCCAGTGCCAGGATCTCAGTT GGCGGTTCATTAGCTGGGTTGGTCGAAGATAGTAAGTGCAATCT	[66]
SL1063	AArGPArPPPAAGGrrPAPPGAGGAAArPr Where r = Pp^1^-dC^2^, P = Nap^3^-dU^4^	[61]

^1^Pp: 5-[N-(phenyl-3-propyl)carboxamide]-2′-deoxy

^2^dC: Deoxycytidine

^3^Nap: 5-[N-(1-naphthylmethyl)carboxamide]-2′-deoxy

^4^dU: Deoxyuridine

Therapeutic methods utilizing monoclonal antibodies (mAbs) to block the PCSK9-LDLR interaction have been established [41]. However, protein-based antagonists such as mAbs have high manufacturing costs due to their large size. Alternatively, strategies focused on reducing PCSK9 expression through lowering its messenger RNA levels using small interfering RNA or antisense oligonucleotides or by introducing loss-of-function mutations into the PCSK9 gene through genome editing using the CRISPR/CRISPR-associated protein system have emerged as alternative approaches to inhibit PCSK9 function. Although these methods have lower manufacturing costs than mAbs, they have certain limitations, including potential off-target effects and the risk of inducing immunity due to the use of a virus vector as a delivery vehicle [67]. Aptamers exhibit significant advantages relative to conventional therapeutics, including their relatively small physical size, flexible structure, quick chemical production, versatile chemical modification, high stability, and lack of immunogenicity [68].

Ando et al. [65] used in vitro aptamer selection (SELEX) to target human PCSK9 and identified a novel PCSK9-binding RNA (PBR) aptamer. They demonstrated that the RNA aptamer exhibits a greater affinity for PCSK9 than for LDLR and effectively inhibits the interaction between PCSK9 and LDLR. Cellular LDL uptake experiments revealed that the RNA aptamer acts as an anti-PCSK9 antagonist by restoring the decrease in LDL uptake caused by PCSK9 in the presence of the PCSK9-binding RNA aptamer. To generate an RNA library, a synthetic DNA library encoding random sequences was subjected to run-off in vitro transcription. The RNA library was then subjected to pull-down assays using PCSK9-nonimmobilized beads followed by human PCSK9-immobilized beads to isolate only the RNAs that bind to PCSK9. The specific binding of the RNA aptamer to PCSK9 was assessed using HRP-based chemiluminescence detection. The chemiluminescence signal was stronger on the PCSK9-immobilized beads than on the control beads, confirming the selective binding of the RNA aptamer to PCSK9. Subsequently, the RNA aptamer was named PBR (PCSK9-Binding RNA). An LDLR pull-down study was conducted to evaluate the inhibitory effect of PBR on the PCSK9-LDLR interaction. The chemiluminescence intensity of LDLR-immobilized beads treated with both PCSK9 and PBR was significantly lower than that of LDLR-immobilized beads incubated with only PCSK9, indicating that PBR impedes the PCSK9-LDLR interaction. PBR exhibited an IC50 value of 120 nM for suppressing PCSK9 binding to LDLR, and at a concentration of 10,000 nM, it almost completely blocked the PCSK9-LDLR interaction. The binding of PBR to PCSK9 was also examined in the presence of Pep2-8, a peptide known to block the PCSK9-LDLR interaction. The qRT‒PCR results indicated that PBR competes with Pep2–8 for binding to PCSK9, as the recovery rate of PBR decreased in the presence of 50 mM Pep2–8. To understand the mechanism of action of PBR against PCSK9, an experiment called “LDLR-dependent cellular LDL uptake” was performed using HepG2 cells. The fluorescence-based LDL uptake assay demonstrated that PCSK9 treatment reduced LDL receptor levels in HepG2 cells, resulting in lower fluorescently tagged LDL uptake. However, when HepG2 cells were pretreated with PBR and then exposed to PCSK9, the fluorescence intensity was comparable to that of cells treated with only PCSK9, indicating that PBR prevents the binding of PCSK9-LDLR in live cells and restores LDL uptake. Furthermore, PBR exhibited significantly lower affinity than did PCSK9-targeting monoclonal antibodies (mAbs), such as evolocumab (KD = 16 pM) [65].

Sattari et al. employed capillary electrophoresis evolution of ligands by exponential enrichment (CE-SELEX) to separate and select aptamers with high affinity for PCSK9. Initially, a randomly selected ssDNA library containing sequences ranging from 10^12^ to 10^16^ was incubated with a low concentration of PCSK9 and subsequently separated using CE. The nonspecific and unbound ssDNA sequences were isolated, while the specific bound ssDNA sequences were retained. The selected precise and bonded sequences were then subjected to PCR amplification. In this study, three rounds of CE-SELEX screening were performed to identify specific aptamers that exhibit strong binding affinity for the PCSK9 protein. Aptamers-1 (AP-1) and − 2 (AP-2) displayed greater thermodynamic stability in their structures than did the other eight aptamers. AP-1 exhibited the greatest binding affinity for PCSK9, with a KD of 294 nM and an exceptionally low IC50 of 325 nM. Similarly, AP-2 demonstrated KD and IC50 values of 323 nM and 327 nM, respectively [66].

Efforts have been made to expand the functional diversity of nucleic acids, either through alterations in the sugar-phosphate backbone or modified nucleobases. One strategy involves introducing an additional base pair with a distinctive hydrogen bonding arrangement to the four-element genetic code [69, 70]. Recent advancements in directed evolution or enzyme engineering techniques have led to the discovery of new polymerases that can generate artificial genetic polymers or xeno nucleic acids with different backbone chemistries not found in natural nucleic acids [71]. These and other ribose modifications, such as L-enantiomer RNA (L-RNA) [72], locked nucleic acids (LNAs) [73], and 2’-amino, 2’-fluoro, and 2’-O-methyl modifications [74, 75], have gained significant attention and application due to their increased resistance to nuclease degradation. Gawande et al. investigated the advantages of enhancing physicochemical diversity by selecting modified DNA aptamers with modifications resembling amino acid side chain changes on both pyrimidine bases for targeting PCSK9 [61]. They explored specific pairs of modifications that could enhance the specificity, blocking efficacy, and metabolic stability of aptamers compared to those with only a single modification. Two modifications were tested on deoxycytidine (dC), namely, Pp and Nap, with unmodified dC serving as a control. Additionally, five alterations were made to deoxyuridine (dU), including Thr, Pp, Moe, Nap, and Tyr, with the original dT serving as a control. A range of hydrophobic aromatic side chains on dC and dU, which are among the most significant single modifications of deoxyuridine, as well as stronger hydrophilic side chains on dU, were tested to identify the optimal combinations for selecting high-affinity ligands. The libraries with two alterations, particularly a hydrophobic modification on dC combined with Tyr-dU (Pp-dC/Tyr-dU and Nap-dC/Tyr-dU), yielded the most promising affinity ligands. Furthermore, the use of a slow off-rate modified aptamer (SOMAmer) with two modifications resulted in improved epitope coverage. The authors evaluated the limits of quantification, precision, accuracy, and linear relationship of plasma dilution to establish a SOMAmer sandwich assay for determining plasma PCSK9 concentrations in human clinical samples. They also determined the plasma concentrations of PCSK9 in clinical specimens and assessed the levels of PCSK9 in HepG2 cells overexpressing PCSK9 in cell-free supernatants. From the Pp-dC/Nap-dU library, an aptamer named SL1063 was discovered that inhibited the entry of fluorescent-tagged LDL into human PCSK9 wild-type and D374Y mutant cells. SL1063 exhibited strong affinity for PCSK9 in rhesus monkeys, mice, and rats. In a fluorescent-tagged LDL uptake reversal assay, this aptamer prevented PCSK9 activity and preserved LDL-R integrity in HepG2 cells treated with wild-type PCSK9. Furthermore, it increased LDL-R expression in a concentration-dependent manner [61].

Ample evidence has demonstrated that PCSK9 can elevate plasma LDL-C levels, contributing to the progression and development of atherosclerosis. Studies have revealed that increased levels of PCSK9 are associated with a greater fraction of necrotic cores in coronary atherosclerosis, independent of LDL-C variation [76]. Furthermore, the measurement of PCSK9 levels has shown promise as a prognostic biomarker for predicting major adverse cardiovascular events (MACE). The baseline serum PCSK9 concentration has been shown to predict the incidence of ASCVD events over a 15-year follow-up period [77]. Additionally, PCSK9 levels have proven to be accurate in predicting acute coronary syndrome (ACS) at a 24-month follow-up in patients with severe carotid artery atherosclerosis undergoing carotid endarterectomy. Specifically, PCSK9 concentrations greater than 431.3 ng/mL have been correlated with a greater risk of ACS occurrence [78]. In patients with coronary artery disease undergoing percutaneous coronary intervention (PCI), baseline PCSK9 levels during a 28.4-month follow-up were associated with MACE and mortality [79]. Consistent with this evidence, among patients with ST-segment elevation myocardial infarction undergoing PCI, those with high PCSK9 levels and diabetes mellitus exhibited the lowest cumulative event-free survival rate [80].

Therefore, the development of accurate methods to detect serum PCSK9 is crucial for monitoring and diagnosing cardiovascular diseases. Advances in biosensor technology have enabled the detection of diseases and tracking of the body’s response to treatment. Notably, nanobiosensors based on aptamers are being extensively investigated as potent analytical tools in clinical analysis [81]. These biosensors offer high sensitivity, rapid response, specificity, portability, simplicity, and reduced cost compared to conventional methods for detecting PCSK9. The use of nanomaterials in the development of aptamer-based biosensors has greatly facilitated the discovery of important and versatile diagnostic devices. Due to their small size, ease of use, nontoxicity, and disposability, aptamer-based biosensors are particularly appealing for clinical analysis [82–85].

Steffen et al. utilized a DNA aptamer-based array to measure the plasma concentrations of various proteins, including PCSK9, in the Atherosclerosis Risk in Communities (ARIC) study [83]. In another study, Lynch et al. employed an aptamer-based proteomic method to assess plasma proteins, including PCSK9, in newborns at risk of retinopathy of prematurity (ROP). They discovered a correlation between clinically severe ROP and plasma PCSK9 levels [84].

Gupta et al. employed magnetic beads along with a biotinylated 56-mer anti-PCSK9 aptamer and a monoclonal antibody to accurately measure the concentration of PCSK9 in plasma. The anti-PCSK9 aptamer was chemically modified with nucleotides (NapdU) that mimic amino acid side chains, leading to enhanced and more specific interactions between nucleic acids and proteins. This modified anti-PCSK9 aptamer was developed as an affinity capture tool for an immunoaffinity-liquid chromatography‒mass spectrometry (IA-LC‒MS) system. A comparative analysis was conducted to assess the effectiveness of the anti-PCSK9 aptamer in comparison to that of a PCSK9 mAb for IA-LC‒MS. Equivalent concentrations of the aptamer and antibody were utilized, and efforts were made to maintain similar sample preparation workflows for both techniques. It was found that comparable molar concentrations of the anti-PCSK9 aptamer were needed to achieve recovery rates similar to those of the monoclonal antibody, underscoring the usefulness of the aptamer reagent in IA-LC‒MS. Notably, due to the use of aptamers consisting of nucleotides rather than amino acids, the trypsin digestion of the eluent resulted in significantly fewer peptides in the LC chromatogram background following immunoaffinity enrichment with the aptamer compared to the mAb [85].

## Conclusion and future perspectives

Inhibition of the PCSK9-LDLR interaction offers a potential approach to mitigate LDLR degradation and reduce plasma LDL-C concentrations. Over the past decade, therapeutic methods utilizing monoclonal antibodies (mAbs) to block the PCSK9-LDLR interaction have been established [41]. However, mAbs have high manufacturing costs due to their large size. Moreover, the use of small interfering RNA or antisense oligonucleotides or the introduction of loss-of-function mutations into the PCSK9 gene through genome editing using the CRISPR/CRISPR-associated protein has certain limitations, including potential off-target effects and the risk of inducing immunity due to the use of a viral vector as a delivery vehicle [67].

Potentially, an alternative approach to these methods involves suppressing the PCSK9-LDLR interaction using RNA aptamers [55, 86]. However, the effectiveness of RNA aptamers may be impacted by nuclease-mediated degradation and renal filtration. Renal filtration can be minimized through a process called PEGylation, which has been utilized in the clinical use of the anti-VEGF RNA aptamer pegaptanib. To protect RNA aptamers from nuclease breakdown, various artificial chemical modifications, such as altering the sugar, base, and phosphate with substances such as 2’-deoxy-2’-fluoro, phosphorothioate, locked nucleic acid, and 3’ and 5’-terminal capping, can be employed [87, 88]. For instance, chemically modifying the sugar of an RNA aptamer with 2’-deoxy-2’-fluoropyrimidine extends its half-life in serum-containing conditions compared to the unmodified aptamer.

Compared with the discovered aptamers, PCSK9-targeting mAbs such as evolocumab exhibit significantly greater affinity. Affinity maturation, a technique commonly used in in vitro selection, can be employed to modify the sequence of aptamers and enhance their effectiveness as antagonists. By combining natural four-base elements with artificial base pairs that are functional during in vitro selection, high-affinity PCSK9-binding RNA aptamers can be developed. Additionally, the use of unnatural bases enables the identification of DNA aptamers with remarkably high affinity for PCSK9 [65, 66]. Aptamers have also been employed as research reagents in protein analysis methods such as flow cytometry, immunoprecipitation, and immunofluorescence. For example, in a mass spectrometry and immunoprecipitation study, an anti-PCSK9 DNA aptamer demonstrated an equivalent detection capability to that of an antibody [67].

The chemical diversity of DNA and RNA aptamers is considerably lower than that of protein-based ligands, which limits their adaptability. Although the addition of unique functional groups to a single base in improved aptamers has significantly improved the success rate of finding nucleic acid ligands for protein targets, the limited chemical diversity of nucleic acid libraries poses challenges in utilizing aptamers for research, diagnosis, and treatment. Natural nucleic acids have fewer building blocks (four bases compared to 20 amino acids) and a polyanionic backbone with more hydrophilic character than protein-based ligands such as antibodies. Moreover, they have a narrow range of functional groups available for target recognition. However, the extensive number of random libraries available for screening partially compensates for this limitation.

Additionally, libraries with two modified nucleotides had a greater number of altered nucleotides, which facilitated the truncation of sequences. Importantly, this increased affinity and improved the encoding of binding domains without compromising specificity. Libraries with two modified nucleotides offer enhanced epitope coverage by identifying ligands that cross-react with PCSK9 from different species and exhibit higher frequency and performance in sandwich pairs [61]. These properties, including specificity, ligand efficiency, high affinity, and species cross-reactivity, are highly desirable for therapeutic development.

To meet the growing demand for medical purposes, further advancements are required in diagnostic tests. The aptamer-based approach holds the potential to become a valuable tool for disease diagnosis, biosensors, and treatment strategies, enabling us to tackle challenges that conventional technologies struggle with. The stability of aptamers in blood and other biological fluids after administration is crucial for their clinical effectiveness. Additionally, the limited ability of aptamers to accumulate intracellularly near the target site restricts their general usage. While aptamers have demonstrated success in in vitro settings, their application in human systems requires careful consideration. Encapsulating aptamers is one approach to protect them from nuclease activity in biological fluids. A reduction in LDL-C is fundamental for the prevention of atherosclerosis and CVD. PCSK9 is a therapeutic target for this purpose, and aptamers may constitute an alternative to available therapies; however, further efforts are still needed to prove this feasibility.

## Data Availability

No datasets were generated or analysed during the current study.
